# Cottonseed protein concentrate as an effective substitute to fish meal in pike perch (*Sander luciperca*) feed: evidence from growth performance and intestinal responses of immune function and microflora

**DOI:** 10.3389/fimmu.2025.1522005

**Published:** 2025-03-04

**Authors:** Ze Fan, Jie Zhao, Jiaming Huang, Cuiyun Lu, Di Wu, Zhipeng Sun, Jinnan Li, Liansheng Wang, Xianhu Zheng

**Affiliations:** ^1^ Key Laboratory of Aquatic Animal Diseases and Immune Technology of Heilongjiang Province, Heilongjiang River Fisheries Research Institute, Chinese Academy of Fishery Sciences, Harbin, China; ^2^ College of Fish and Life Science, Shanghai Ocean University, Shanghai, China; ^3^ Key Laboratory of Aquatic, Livestock and Poultry Feed Science and Technology in South China, Ministry of Agriculture and Rural Affairs, Guangdong Evergreen Feed Industry Co., Ltd., Zhanjiang, China

**Keywords:** pikeperch (*Sander luciperca*), cottonseed protein concentrate (CPC), 60% substitution ratio for fish meal, decreased *igf1* expression, intestinal microflora

## Abstract

**Introduction:**

The aim of this study was to assess the response characteristics of intestinal immune function and microflora of pike perch (Sander luciperca) receiving cottonseed protein concentrate (CPC) as a substitute for fishmeal.

**Methods:**

A basal diet was formulated to contain 55% fish meal, and then CPC was used to replace 0% (CPC0), 20% (CPC1), 40% (CPC2), and 60% (CPC3) of dietary fish meal. The four diets were fed to pikeperch with an initial body weight of 3.55 ± 0.01 g for 8 weeks.

**Results:**

The results revealed that there were no significant effects of dietary CPC levels on survival rate, mass gain rate, and specific growth rate. The highest value for the feeding efficiency ratio was found in the CPC3 group and was significantly higher than that of the control group. Significantly lower values for the hepatosomatic index, crude ash content, and higher crude protein content were observed in the CPC3 group. Trypsin activity in the CPC3 group was lowest compared to the control group and CPC1 group. Among the three CPC substitution groups, increases in intestinal antioxidant enzyme activities, glutathione content, and anti-inflammatory factor expression, and downregulation of pro-inflammatory factors were observed with increasing CPC substitution. The injury degree of the intestinal mechanical barrier was mitigated along with relief of oxidative damage and inflammation with increasing CPC substitution ratios.

**Discussion and conclusion:**

In conclusion, 60% of fish meal can be replaced by CPC based on the above results. However, increasing dietary CPC substitution slightly increased Firmicutes abundance and significantly decreased Actinobacteriota abundance, but the abundance of Proteobacteria in the CPC3 group was appreciably increased. An increase in Staphylococcus and a reduction of Lactobacillus were observed in the CPC2 and CPC3 groups. Igf1 expression was significantly downregulated with increasing CPC substitution. Henceforth, the above two limiting factors should be considered key breakthroughs in improving the effectiveness of replacing fish meal with CPC in pikeperch. The current findings provide a significant reference and breakthrough in improving the effectiveness of replacing fishmeal with CPC in pikeperch diets.

## 1 Introduction

Pikeperch (*Sander luciperca*), which belongs to *Perciformes*, *Percidae*, *Lucioperca*, is one of the most potentially commercial fishes in the *Percidae* family (1). With its tender meat, strong disease resistance, and high nutritional value, pikeperch has gained high market recognition in Europe and is one of the freshwater fish with great development potential in the aquaculture industry (2, 3). In the early 1960s, it spread from Central Asia to the Ili River system and the Irtysh River system in Xinjiang, and was also introduced in the Heilongjiang River system to release its flow, thus settling in the lower Heilongjiang River, the Wusuli River and Xingkai Lake (4). Whereafter, the artificial breeding of pikeperch in China achieved success in the 1990s (5). At present, pikeperch is widely cultured in the northeast, northwest, North China, East China, South China, and other areas, and has been deemed one of the most promising and marketable farmed fish species in European and Chinese inland aquaculture (6). To satisfy the production demands and boost cultural benefit, the research and development of cost-effective feed for pikeperch has been in urgent need of tackling. Numerous existing studies have proposed that pikeperch has a high protein requirement with an optimal protein level of 47% to 57.7% (7–9), which is higher than 39.9% to 51% for largemouth bass (*Micropterus salmoides*) in the same *Perciformes* family (10).

The use of high-quantity and high-quality fish meal is currently the mainstream solution for dealing with the high protein requirements of pikeperch (7–9). However, almost all feed formulations for carnivorous fish with high protein requirements add a high proportion of fish meal to achieve an effective protein supply (11). However, the aquaculture industry has to face the worrying fact that a significant but declining proportion of world fisheries production is processed into fish meal on account of the availability of natural sources, the deterioration of the marine environment, and the El Niño-Southern Oscillation (12). There is an urgent need to transform the selection of feed protein sources to cope more effectively with the contradiction between fishmeal supply and demand. Hence, plant protein sources to replace fish meal are widely studied and evaluated to accomplish the protein requirement of farmed species in *Perciformes*, *Percidae* such as European seabass (*Dicentrarchus labrax*) (13) and spotted seabass (*Lateolabrax maculatus*) (14) and largemouth bass (15). Nevertheless, there is a lack of relevant attempts in the study of pikeperch.

The exploration of plant protein sources should take full consideration of economic benefits, ecological benefits, and sustainable development of the pikeperch industry. As a new breakthrough, cottonseed protein concentrate (CPC) is obtained by extracting cottonseed flakes at low temperatures to prevent protein denaturation and using mixed extraction solvent to reduce the free gossypol content. In comparison with cottonseed meal (CSM), CPC possesses higher crude protein content (> 60%), more reasonable amino acid composition (particularly arginine, leucine, and valine), and especially low FG content (< 400 mg/kg). Despite the advantages of CPC, there are still some potential allergens (vicilin A and vicilin C72, etc.) for aquatic animals that cannot be ignored. Different aquatic animals have different tolerances to free gossypol and other allergens, thus CPC should be applied for different aquatic animals as appropriate (16). Additionally, CPC has higher carbohydrate content than fish meal, and consequently, overuse of CPC will impose a burden on carbohydrate metabolism and the insulin signaling pathway (17), thereby weakening immunity. Up to now, a certain amount of CPC has been reported to replace dietary fish meal in a variety of farmed species in *Perciformes* without impacting the growth performance, including golden pompano (*Trachinotus ovatus*) (18), largemouth bass (19), and large yellow croaker (*Larimichthys crocea*) (20). Furthermore, Liang et al. (21) reported that CPC, as a substitute for fish meal in the high-protein aquatic feed of sea perch (*Lateolabrax japonicus*), significantly improved the radial expansion degree of the expanded feed and reduced the particle bulk weight, which was conducive to the processing of floating feed. Overall, the application and study of CPC as a novel plant protein source to replace fish meal in pikeperch has not been implemented to date.

In fish, the intestine performs irreplaceable and vital functions in the immunologic barrier, nutrient absorption, and resistance to invasion by pathogens and bacterial toxins in the internal environment (22). Therefore, intestinal health is regarded as an extremely critical target in the evaluation of novel protein source applications for farmed fish (23, 24). Existing studies have shown that intestinal adaptation to different proportions of CPC varies to a certain extent. An appropriate CPC proportion has positive effects on intestinal immunity, digestive enzyme activity, and intestinal flora in different farmed fish (18, 19). Excessive substitution ratios can have negative effects, as it has been reported that excessive replacement of fish meal with CPC caused a decrease in digestive enzyme activity in sturgeon (*Acipenser schrenckii*) (25) and impaired the intestinal health of and caused intestinal inflammation in hybrid grouper (♀*Epinephelus fuscoguttatus*×♂*Epinephelus lanceolatu*) (26). These reports prompted us to concentrate on the responses of the intestine when assessing the potential of CPC to replace fish meal in pikeperch feed.

In consideration of existing foundation and development prospects, this study aims to explore the utilization of CPC as a fish meal replacer in the diets of pikeperch, focusing on its impact on growth performance, immune and digestive indexes, intestinal morphology, and intestinal microflora. By evaluating these parameters, this research seeks to provide a theoretical basis for curtailing the amount of fish meal in commercial pikeperch feed and accelerating the green and healthy development of the pikeperch aquaculture industry.

## 2 Materials and methods

All animal procedures in this study were conducted according to the guidelines for the care and use of laboratory animals of Heilongjiang River Fisheries Research Institute (CAFS). The studies in animals were reviewed and approved by the Committee for the Welfare and Ethics of Laboratory Animals of CAFS.

### 2.1 Design and preparation of experimental diets

In this study, cottonseed protein concentrate purchased from Xinjiang Jinlan Plant Protein Co., Ltd (Shihezi, China) was obtained through a series of processes including mild temperature extraction, depigmentation, and controlled reduction of carbohydrate content from cottonseed meal (16). CPC is characterized by 61.51% crude protein, 2.36% crude lipid, 0.85% methionine, 2.47% lysine, and 230 mg/kg free gossypol.

Four isoproteic and isolipidic diets supplemented with CPC, substituting either 0%, 20%, 40%, or 60% of fishmeal (FM) protein, were designed and applied in the four experiment groups, named CPC0, CPC1, CPC2 and CPC3, respectively. The formulated dietary crude protein level (54%) and crude lipid level (12%) were the same as those in commercial feeds and the study of Zhao et al. (9). The formulation and proximate composition of the experimental diets are exhibited in **Table 1**. The feed production process was consistent with previous studies in our laboratory (9, 27). The manufactured 1.5 mm pellets were stored in a drying oven at 60 °C for 3 h, and then naturally air dried. The dietary pellets were kept at -20 °C for later trials.

**Table 1:** Formulation and proximate composition of the experimental diets (dry matter basis, DM%).

Ingredient	CPC0	CPC1	CPC2	CPC3
Fish meal	55.00	44.00	33.00	22.00
Cottonseed protein concentrate	0.00	11.00	22.00	33.00
Chicken powder	5.00	5.00	5.00	5.00
Corn gluten meal	6.00	6.00	6.00	6.00
Soy protein concentrate	8.50	8.50	8.50	8.50
Wheat gluten	4.00	4.20	4.40	4.60
Wheat flour	6.50	7.50	8.50	9.38
Fish oil	5.20	5.60	6.00	6.40
Lecithin oil	2.50	2.50	2.50	2.50
Premix V+M ^a^	1.00	1.00	1.00	1.00
Choline chloride	0.50	0.50	0.50	0.50
Cellulose	4.78	3.18	1.58	0.10
Sodium alginate	1.00	1.00	1.00	1.00
Butylated hydroxytoluene (BHT)	0.02	0.02	0.02	0.02
Moisture	6.37	6.64	6.49	6.69
Crude protein	54.11	54.15	54.16	54.19
Crude lipid	12.05	12.06	11.96	12.01
Crude ash	10.70	10.62	10.60	10.55

^a^ Premix-V + M (mg/kg or IU/kg diet): Vitamin A, 750,000 IU; Vitamin D_3_, 200,000 IU; Vitamin E, 6,000 mg; Vitamin K_3_, 2,000 mg; Vitamin B_1_, 1,200 mg; Vitamin B_2_, 1,200 mg; Vitamin B_12_, 8 mg; Vitamin C, 21,000 mg; D-calcium pantothenate, 2,000 mg; niacinamide, 9,000 mg; folic acid, 370 mg; D-biotin, 15 mg; inositol, 10,000 mg; MgSO_4_, 6,000 mg; ZnSO_4_, 4,000 mg; MnSO_4_, 2,500 mg; CuSO_4_, 2,500 mg; FeSO_4_, 2,500 mg; CoSO_4_, 160 mg; Ca(IO_3_) 2, 200 mg; and Na_2_SeO_3_, 40 mg.

### 2.2 Pikeperch feeding trial

Pikeperch were sourced from the Hulan Experimental Station, Heilongjiang Fisheries Research Institute (Harbin, China). After transportation to the recirculating aquaculture operation room of the Heilongjiang Fisheries Research Institute, the fish were fed with an even mixture of the four diets for 2 weeks to adapt to the experimental diets and culture environment. Through the 2-week acclimation, a total of 360 juvenile pikeperch (3.55 ± 0.01 g) were selected and randomly distributed into 12 indoor fiberglass aquariums with a length, wide, height, and water capacity of 0.6 m, 0.45 m, 1.0 m, and 270 L, respectively. Three replicates (aquariums) were arranged per treatment and each aquarium contained 30 fish.

The feeding experiment was executed in the water-recirculating aquaculture facilities of the Fish Nutrition and Feed Department with a feeding period of 8 weeks. Throughout the 8-week experiment, all the fish were hand-fed twice daily at 7:00 and 17:00 to apparent satiation. The residual feeds and feces waste were siphoned out by observing the food intake, and one-third of the circulating water was renewed twice a week. During the feeding trial, an air compressor was utilized to guarantee that the dissolved oxygen in the aquarium was above 6 mg/L. Furthermore, the water temperature was maintained at 23 ± 1°C, pH was 7.7 to 7.9, and the natural photoperiod was implemented.

### 2.3 Calculation of growth indicators and morphological parameters

When the feeding trial was finished, all the pikeperch were counted and weighed together after 24 hours of fasting to calculate the survival rate (SR), mass gain rate (MGR), specific growth rate (SGR), and feeding efficiency ratio (FER). Six fish per aquarium were randomly selected and anesthetized with MS-222 (Ethyl m-aminobenzoate mesylate, 100 mg/L) (28) to individually measure body mass, body length, visceral mass, and liver mass to calculate the condition factor (CF), viscerosomatic index (VSI), and hepatosomatic index (HSI), respectively.

### 2.4 Sample acquisition for subsequent experiments

After collecting the growth data, the aforementioned (in section 2.3) six chosen fish per aquarium were dissected to obtain the intact intestine samples and intestinal contents. Of the six intestine samples, four of them were fast-frozen in liquid nitrogen, and stored at -80°C for RNA extraction and enzyme activity measurement. For the other two intestine samples, anterior intestines were fixed with 4% paraformaldehyde fixative to observe the tissue structure. The intestinal contents were collected with two biological replicates in each aquarium, and the intestinal contents of three pikeperch were mixed as a sample for each biological replicate. Then, the samples were fast-frozen in liquid nitrogen and stored at −80 °C for analysis of intestinal microflora composition. Furthermore, six fish were randomly selected from each aquarium and stored at -20 °C for proximate body composition.

### 2.5 Index determination method

#### 2.5.1 Proximate body composition

The proximate compositions of the experimental diets and whole fish were detected using the Association of Official Agricultural Chemists’ methods (29). The methods mainly consisted of the atmospheric drying method for moisture, the Kjeldahl method for crude protein, the Soxhlet method for crude lipid, and the combustion method for crude ash.

#### 2.5.2 Activities evaluation of digestive and antioxidant enzymes in the intestine

The enzyme activities of trypsin (A080-2-2), lipase (LPS, A054-1-1), amylase (AMS, C016-1-1), superoxide dismutase (SOD, A001-3-2), and catalase (CAT, A007-2-1); total antioxidant capacity (T-AOC, A015-2-1); the total protein (TP, A045-2-2); and reduced glutathione (GSH, A006-2-1) contents in the intestine were estimated using commercial kits (Jian Cheng Bioengineering Institute, Nanjing, China).

#### 2.5.3 Observation of intestinal tissue morphology

Paraformaldehyde-fixed intestine tissues were dehydrated with gradient grades of ethanol, followed by embedding with paraffin, according to the method of Hao et al. (30). These paraffin blocks were cut into 5 μm sections with a slicer (Leica RM2235, Heidelberg, Germany), which were stained with hematoxylin-eosin (Shaanxi Eco Biotechnology Service Co., LTD, Xianyang China) dye (HE) to produce tissue sections. A light microscope (Leica MD 2000B) was used for follow-up morphological observation.

#### 2.5.4 Intestinal gene expression quantification

The total RNA extraction from the intestine samples was conducted according to the manufacturer’s instructions of RNAiso Plus [Takara Biomedical Technology (Dalian) Co., Ltd]. To ensure the purity and quality of the isolated RNA, quantitative OD260/280 were analyzed by spectrophotometry at 260 and 280 nm respectively, and the quality of the RNA was verified using the agarose gel electrophoresis.

The TaKaRa PrimeScriptTM RT reagent Kit with gDNA Eraser (Perfect Real Time) (Code No: RR047A), provided by Takara Biomedical Technology (Dalian) Co., Ltd., was applied to synthesize the first-strand cDNA, and the obtained cDNA was stored at -20°C for the subsequent quantitative polymerase chain reaction (qPCR).

The specific reaction mixture, thermal cycle program, and expression calculation method for the qRT-PCR reaction were performed according to our previous study (31). The qPCR was performed using the Applied Biosystems 7500 real-time PCR system. The reaction was conducted in a 20-μl volume containing 10 μl SYBR^®^ Premix DimerEraser (2×), 0.4 ul PCR Forward Primer, PCR Reverse Primer, 2 μl cDNA template (≈100 ng), and 7 μl RNase free dH_2_O according to the manufacturer’s protocol for the TaKaRa SYBR^®^ Primix Ex TaqTM (Tli RNaseH Plus) (Code No: RR420A) (Dalian Takara Company, Dalian, China). The primers in **Table 2** were derived from the obtained sequences from the NCBI. For normalization, glyceraldehyde-phosphate dehydrogenase (GAPDH, GenBank accession: XM_031299603) in the hepatopancreas was selected as the internal standard on the account of its stable expression. The 2^-ΔΔCt^ method was used to determine the relative expression levels of each gene.

**Table 2:** Specific primers for the qPCR in this research.

Target gene	Accession number	Primer sequence (5’→3’)		Annealing temperature(°C)	Product length	GC (%)
*mtor*	XM_031286196.2	F	CTCCCAGTCCAACCAAGGAC	60.9	165	60
		R	CGTTCACCTCAGGCCAATGA			55
*akt1*	XM_031279117.2	F	GAGATGATGTGCGGCAGACT	59.9	90	55
		R	CCGAGGAAAGCGGATGTCTT			55
*rps6k1*	XM_031278705.1	F	CCATTGCCCTCTCAGGGATG	60.9	90	60
		R	GACTCAGCAATTCGCAAGCC			55
*eif4ebp*	XM_031306363.2	F	TCGTCAGTTTAGCGAGAGCA	58.9	193	50
		R	TGGGGCAGTCTGAGCAATAG			55
*ghr*	XM_031305905.2	F	GGAGAGCACCTTAGCCACAGAG	60.1	141	50
		R	GTGGTGTAGCCGCTTCCTTCT			50
igf1	XM_031313442.2	F	CTGTGCACCTGCCAAGACTA	58.9	115	55
		R	TGTGCCCTTGTCCACTTTGT			50
igf2	XM_031282902.2	F	AAGACACGGACACCACTCAC	59.9	142	55
		R	TGCCGAGGCTATTTCCACAG			55
*claudin-15a*	XM_031289825.2	F	CGAACCGTTACTGGAGGACC	59.9	169	60
		R	AAGGCACGAGACGCTTGAAT			50
*occludin-a*	XM_035991813.1	F	ATCATCTGCGCCATCCTAGC	59.9	191	50
		R	TCCAGGACGCAGTAGTGGTA			50
*zo-2*	XM_031300456.2	F	ACTGGCCTCTTATCCGAGCA	59.9	187	55
		R	CAGCACGTCTGACACGATGA			55
*il1-β*	XM_031287947	F	TGCAGTCTGTGGCTAACCTG	59.9	159	55
		R	TTCTCTTCACGCCTGTGGAC			55
*il8*	XM_031286001.2	F	GATGAGTCTGAGAAGCCTGGG	60.9	122	57.1
		R	TCTCGTCGCAATGAGAGTTGG			52.4
*tnf-β*	XM_031313322.2	F	TGGCCCTTTGTTTAGGAGGC	59.9	82	55
		R	GTCTGGCCTGGTTGTGTCAT			55
*tgf-β*	XM_031285154.2	F	TTTTGGCCCTGTACCAGCAT	58.9	102	50
		R	GCCTGCCCACGTAATAGAGG			60
*gapdh*	XM_031299603	F	GGACCCATGAAGGGCATTCT	59.9	243	55
		R	TGGCATAGTGCAGTGACGAG			55

mechanistic target of rapamycin kinase (*mtor*), AKT serine/threonine kinase 1 (*akt1*), ribosomal protein S6 kinase B1 (*rps6k1*), growth hormone (*gh*), insulin-like growth factor 1 (*igf-1*), insulin-like growth factor 2 (*igf-2*), eukaryotic initiation factor 4E binding protein (*eif4ebp*), interleukin 1 β (*il1-β*), interleukin 8 (*il8*), tumor necrosis factor β (*tnf-β*), transforming growth factor β (*tgf-β*), zonula occludens-2 (*zo-2*).

#### 2.5.5 Microflora analysis of the intestine

The intestinal microflora samples were transported to Shanghai Majorbio Bio-pharm Technology Co., Ltd. Shanghai, China to conduct the microflora analysis of the intestine samples. The detailed operating rules and analytical procedures were as described in our previous study (9). Total microbiota genomic DNA was separated from the intestinal contents of pikeperch with the E.Z.N.A. DNA Kit (Omega Bio-tek, Norcross, GA, USA). An ABI GeneAmp 9700 PCR thermocycler (ABI, Los Angeles, CA, USA) was used to amplify the V3-V4 variable regions of the bacterial 16S rRNA gene. Next, paired-end sequencing of purified amplicons was carried out using the Illumina MiSeq PE300 platform (Illumina (China) SCIENTIFIC Equipment Co., Ltd., Shanghai, China).

### 2.6 Data analyses

All data were analyzed using the statistical software SPSS 20.0 (SPSS Inc., Chicago, IL, USA). The data were subjected to a one-way analysis of variance (ANOVA). When there were significant differences (*P* < 0.05), the group means were further compared using Duncan’s multiple-range test. The difference was viewed as significant when *P* < 0.05. The experimental data were presented as the means ± standard deviation (means ± SD).

## 3 Results and analysis

### 3.1 Effects of substituting fish meal with CPC on pikeperch growth

As shown in **Table 3**, there were no significant effects of dietary CPC levels on SR, MGR, and SGR (*P* > 0.05), but the lower values of MGR and SGR in the CPC substitution groups were observed compared to the CPC0 group. With regard to the CPC substitution groups, the values of MGR and SGR tended to increase with increasing CPC substitution ratios (*P* > 0.05). In contrast, FER among all groups tended to be significantly elevated, and the highest FER was observed in the CPC3 group (*P* < 0.05).

**Table 3:** Growth performance of pikeperch (*Sander luciperca*) fed with increasing substitution ratios of cottonseed protein concentrate (CPC).

Item	CPC0	CPC1	CPC2	CPC3
IBM/g	3.56 ± 0.00	3.54 ± 0.01	3.56 ± 0.01	3.55 ± 0.01
FBM/g	24.33 ± 0.82	22.25 ± 1.70	22.23 ± 1.78	23.37 ± 1.41
SR/%	98.15 ± 0.93	95.37 ± 0.93	100.00 ± 0.00	96.30 ± 2.45
MGR/%	577.38 ± 27.13	534.29 ± 44.96	537.74 ± 42.26	550.83 ± 29.73
SGR/(%/day)	3.41 ± 0.07	3.29 ± 0.12	3.30 ± 0.11	3.34 ± 0.09
FER	0.83 ± 0.02^A^	0.89 ± 0.02^AB^	0.92 ± 0.01^AB^	0.96 ± 0.04^B^

Survival rate (SR) (%) = 100 × (final number of fish/initial number of fish); Mass gain rate (MGR) (%) = 100 × [final mass (g) - initial mass(g)]/initial mass (g); Specific growth rate (SGR) (%/day) = [ln (final mass) - ln (initial mass)]/days of feeding trial × 100; Feeding efficiency ratio (FER) = weight gain (g)/feed intake (g).

Among the values in each row, different capital letter suffixes represent the degree of data difference (*P* < 0.05), and n = 3.

### 3.2 Morphological indexes and proximate body composition

As listed in **Table 4**, there was no significant difference in the values of VSI and CF and the moisture content and crude lipids between the substitution groups and CPC0 group (*P* > 0.05). When compared to the CPC0 group, the HSI value in the CPC2 and CPC3 groups and crude ash content in the CPC3 group was significantly decreased (*P* < 0.05), while the crude protein content in the CPC1 group was markedly decreased (*P* < 0.05).

**Table 4:** Morphological indexes and proximate body composition of pikeperch (*Sander luciperca*) fed with increasing substitution ratios of cottonseed protein concentrate (CPC).

Item	CPC0	CPC1	CPC2	CPC3
HSI/%	1.91 ± 0.05^B^	1.68 ± 0.14^AB^	1.49 ± 0.10^A^	1.51 ± 0.11^A^
VSI/%	14.58 ± 0.55	14.44 ± 0.37	13.73 ± 0.66	14.86 ± 0.72
CF/(g/cm^3^)	0.90 ± 0.02	0.81 ± 0.06	0.88 ± 0.01	0.86 ± 0.01
Moisture/%	72.31 ± 0.34	72.30 ± 0.13	72.82 ± 0.14	73.06 ± 0.41
Crude protein/%	17.53 ± 0.40^B^	16.32 ± 0.59^A^	16.90 ± 0.18^AB^	16.98 ± 0.21^AB^
Crude lipid/%	25.16 ± 0.48	26.76 ± 0.96	26.93 ± 0.34	27.15 ± 1.09
Crude ash/%	11.39 ± 0.09^B^	11.83 ± 0.10^B^	11.16 ± 0.12^B^	9.72 ± 0.08^A^

Condition factor (CF) (g cm^-3^) = 100 × [final body mass (g)/body length (cm)^3^]; Viscerosomatic index (VSI) (%) = 100 × [final visceral mass (g)/final body mass (g)]; Hepatosomatic index (HSI) (%) = 100 × [final liver mass (g)/final body mass (g)).

Among the values in each row, different capital letter suffixes represent the degree of data difference (*P* < 0.05), and n = 3.

### 3.3 Digestive features of the intestine samples

**Figure 1:**
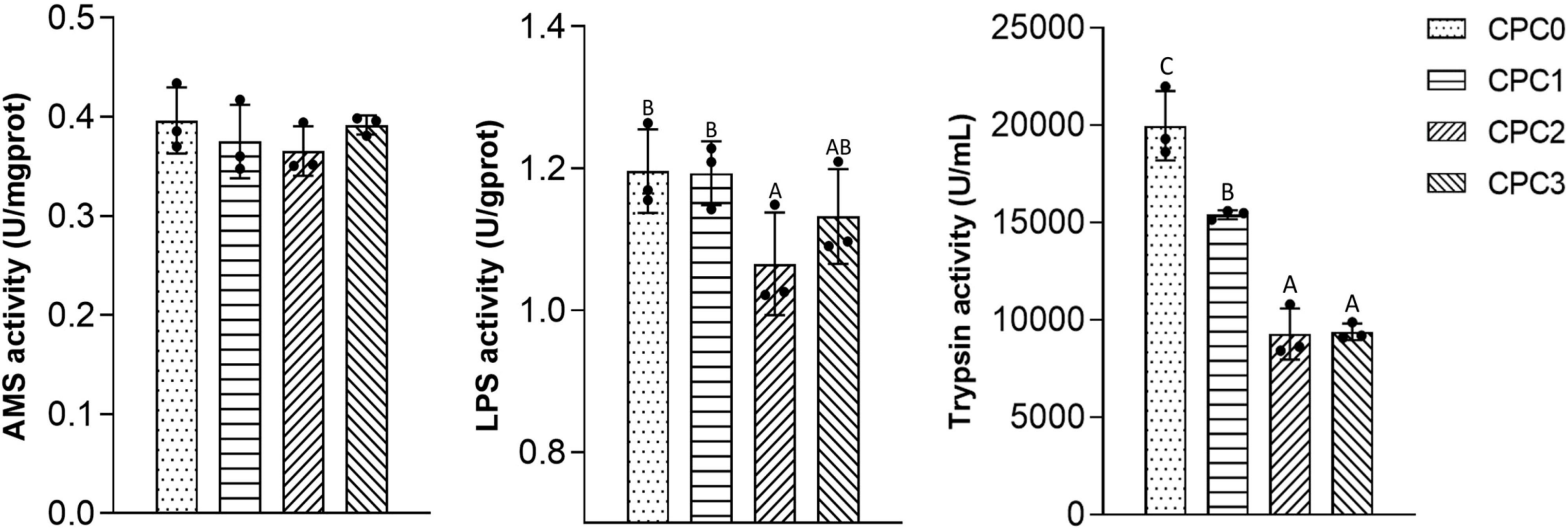
Digestive features of the intestines of pikeperch (*Sander luciperca*) fed with increasing substitution ratios of cottonseed protein concentrate (CPC). Among the values in each column, different capital letter labels represent the degree of data difference (*P* < 0.05), and n = 3. Lipase (LPS), amylase (AMS).

The analysis results of intestinal digestive enzyme activities in the pikeperch are presented in **Figure 1**. For AMS activity, no significant differences were observed among all the treatments (*P* > 0.05). The activity of LPS in the CPC2 group was significantly lower than that in the CPC0 group. Trypsin activity was significantly decreased when the dietary CPC replacement level was above 40% (the CPC2 and CPC3 groups) (*P* < 0.05).

### 3.4 Antioxidant features of the intestine

**Figure 2:**
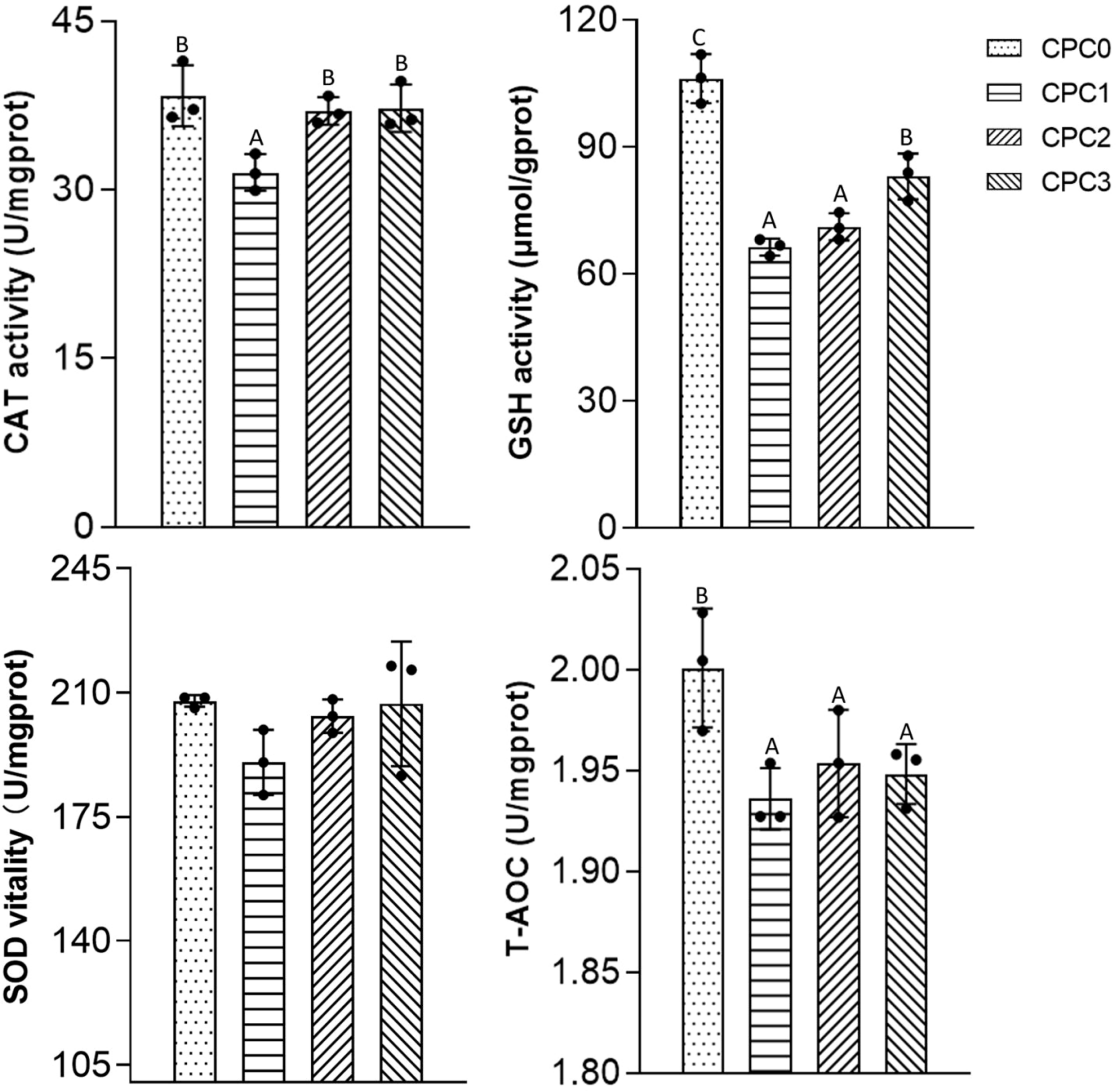
Antioxidant features of the intestines of pikeperch (*Sander luciperca*) fed with increasing substitution ratios of cottonseed protein concentrate (CPC). Among the values in each column, different capital letter labels represent the degree of data difference (*P* < 0.05), and n = 3. Superoxide dismutase (SOD), catalase (CAT), total antioxidant capacity (T-AOC), glutathione (GSH).

As shown in **Figure 2**, the activities of CAT, SOD, and GSH and T-AOC content tended to decline in the CPC substitution groups compared to the CPC0 group. Therein, significantly lower GSH activity and T-AOC content were observed in the CPC substitution groups (*P* < 0.05), and significantly lower CAT activity was observed in the CPC1 group (*P* < 0.05). In terms of the CPC substitution groups, both CAT and GSH activity in the CPC3 group were markedly higher in comparison to the CPC1 group (*P* < 0.05), whereas there was no significant difference in the CAT and SOD activities between the CPC3 group and CPC0 group (*P* > 0.05).

### 3.5 Intestinal morphological features

**Figure 3:**
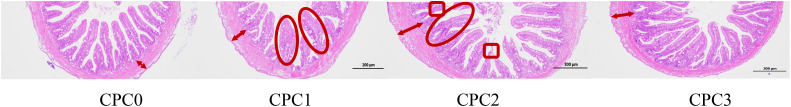
Changes in the morphological features of mid-intestines of pikeperch (*Sander luciperca*) fed with increasing substitution ratios of cottonseed protein concentrate (CPC). Double-headed arrows represent the muscle layer thickness. Ellipses represent the confluent intestinal villi. Boxes represents the villi vacuolation.

As shown in **Figure 3**, the HE-stained sections illustrated that dietary CPC substitution appeared to result in poor intestinal morphology for pikeperch. In general, CPC substitution thickened the muscle layer to a certain extent, especially in the CPC3 group according to the HE-stained sections. Additionally, more confluent intestinal villi and more obvious villi vacuolation were observed in the CPC1 and CPC2 groups compared to the CPC0 and CPC3 groups. More particularly, the intestine samples in the CPC3 group had exposed intestinal villi. The CPC3 group had fewer intestinal villi compared to the CPC0 group.

The CPC3 group had significantly increased villus height by approximately 16.37% and 28.80% compared to the CPC0 and CPC1 groups, respectively (*P* < 0.05). The CPC3 group had significantly decreased villus width by approximately 26.55% compared to the CPC2 group (*P* < 0.05). No significant difference in layer thickness was observed among all treatments (*P* > 0.05).

### 3.6 Intestinal gene expression features

**Figure 4:**
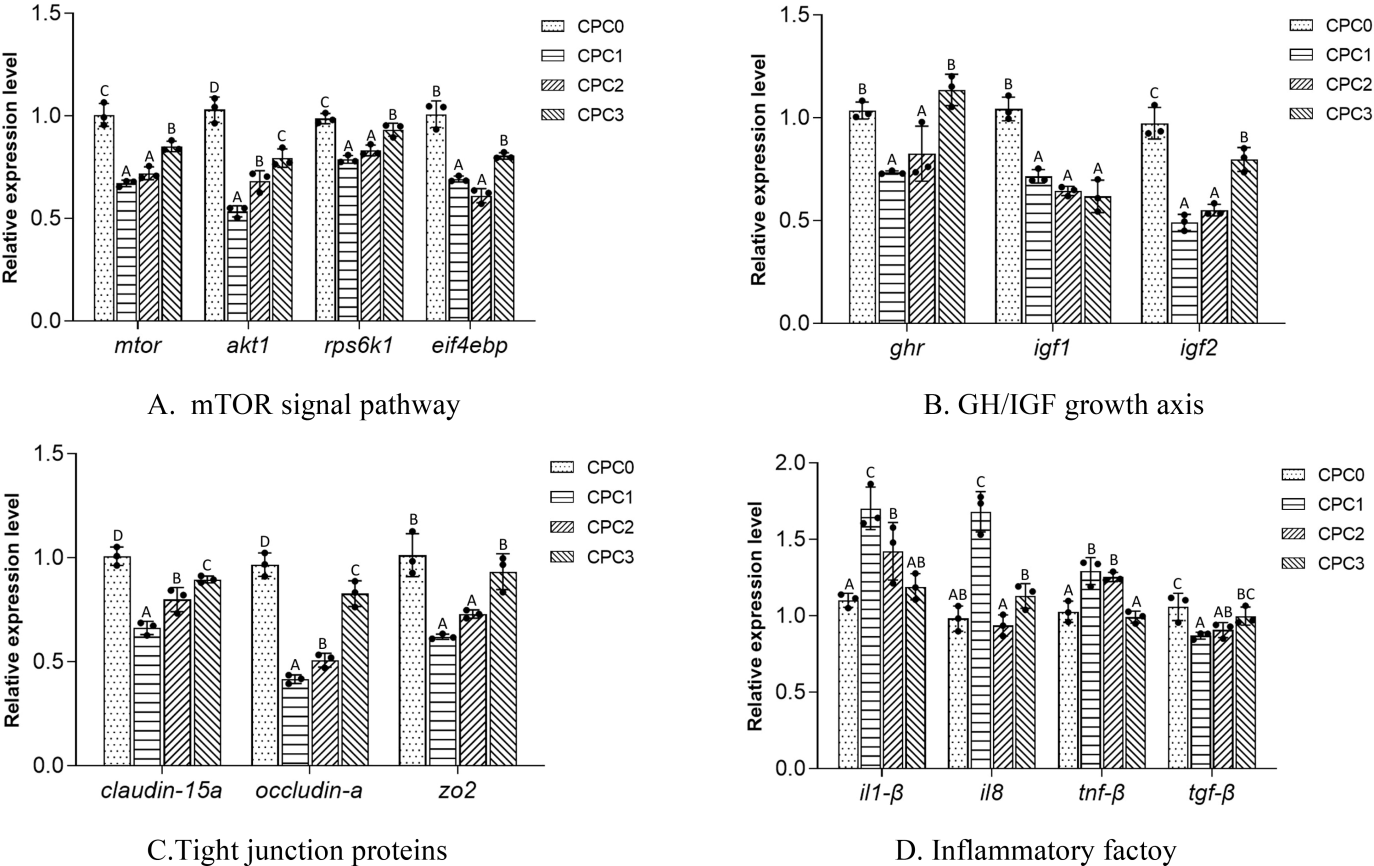
**(A-D)** Intestinal gene expression features in pikeperch (*Sander luciperca*) fed with increasing substitution ratios of cottonseed protein concentrate (CPC). Among the values in each column, different capital letter labels represent the degree of data difference (*P* < 0.05), and n= 3. Mechanistic target of rapamycin kinase (*mtor*), AKT serine/threonine kinase 1 (*akt1*), ribosomal protein S6 kinase B1 (*rps6k1*), growth hormone (*gh*), insulin-like growth factor 1 (*igf-1*), insulin-like growth factor 2 (*igf-2*), eukaryotic initiation factor 4E binding protein (*eif4ebp*), interleukin 1 β (*il1-β*), interleukin 8 (*il8*), tumor necrosis factor β (*tnf-β*), transforming growth factor β (*tgf-β*), zonula occludens-2 (*zo-2*).

The gene expressions in the intestine of pikeperch in the different dietary CPC substitution groups are presented in **Figure 4**. In sum, the intestinal expressions of genes related to the mTOR signal pathway, GH/IGF growth axis, tight junction proteins, and anti-inflammatory cytokine (*tgf-β*) were significantly downregulated to varying degrees in the CPC-supplemented groups (especially in the CPC1 and CPC2 groups) compared to the CPC0 group (*P* < 0.05), while pro-inflammatory cytokines (*il-1β*, *il8*, *tnf-β*) were significantly upregulated to varying degrees. No significant difference in the intestinal expression of *eif4ebp*, *ghr*, *zo2*, *il-1β*, *il8*, and *tnf-β* was observed between the CPC0 and CPC3 groups (*P* > 0.05). From the perspective of the CPC-supplemented groups, a majority of the intestinal expressions of genes, except *igf1* and *tnf-β* in the CPC3 group, were significantly upregulated compared to those in the CPC1 and CPC2 groups (*P <*0.05).

### 3.7 Description of changes in intestinal microflora

#### 3.7.1 Analysis of microflora diversity

**Figure 5:**
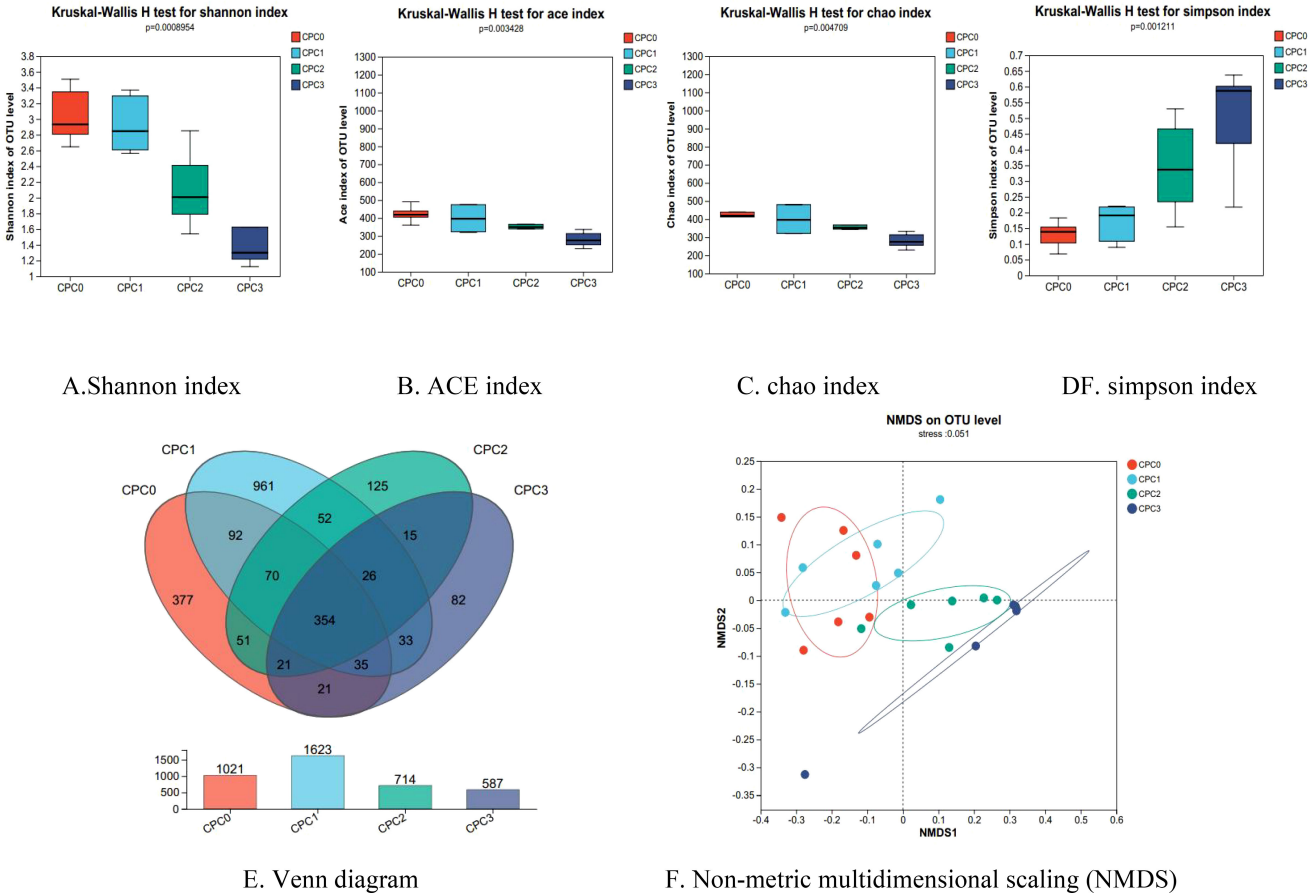
**(A-F)** Alpha diversity analysis and Beta diversity analysis of intestinal microflora in pikeperch (*Sander luciperca*) fed with increasing substitution ratios of cottonseed protein concentrate (CPC).

The Shannon curve tended to flatten out and the coverage exceeded 99.9%, indicating that the detection rate of microflora communities in the intestine content samples was close to saturation. Currently, sequencing can cover the vast majority of species in the samples (**Supplementary Figures S1A, B**). **Figures 5A–D** show the relationship between the CPC substitution ratio and alpha diversity. Both microflora community richness indices (ACE and chao) and community diversity indices (Shannon and Simpson) negatively responded to the dietary CPC substitution ratio. Increases in the dietary CPC substitution ratio significantly decreased the ACE, chao, and Shannon indices and increased the Simpson index.

**Figures 5E, F** show that after comparing the overall structure of the community through beta diversity, the community composition difference was further explored. The predominant operational taxonomic units (OTUs) among the CPC0 and CPC substitution groups was assessed by drawing a Venn diagram using the 16S rRNA sequencing data to further identify the core and different OTUs existing in different groups. As presented in **Figure 5E**, 354 OTUs were shared among all the intestine content samples. In contrast, 377, 961, 125, and 82 OTUs were unique to the CPC0, CPC1, CPC2, and CPC3 groups, respectively. The CPC3 group had the lowest number of unique OTUs whereas the CPC1 group had the highest number of unique OTUs. Non-metric multidimensional scaling (NMDS) analysis of the microflora communities was applied to evaluate whether the changes in community structure were significant. The NMDS analysis illustrated that the degree of separation of the microflora composition tended to be more obvious with increases in the dietary CPC substitution ratio.

#### 3.7.2 Features of microflora community composition

**Figure 6:**
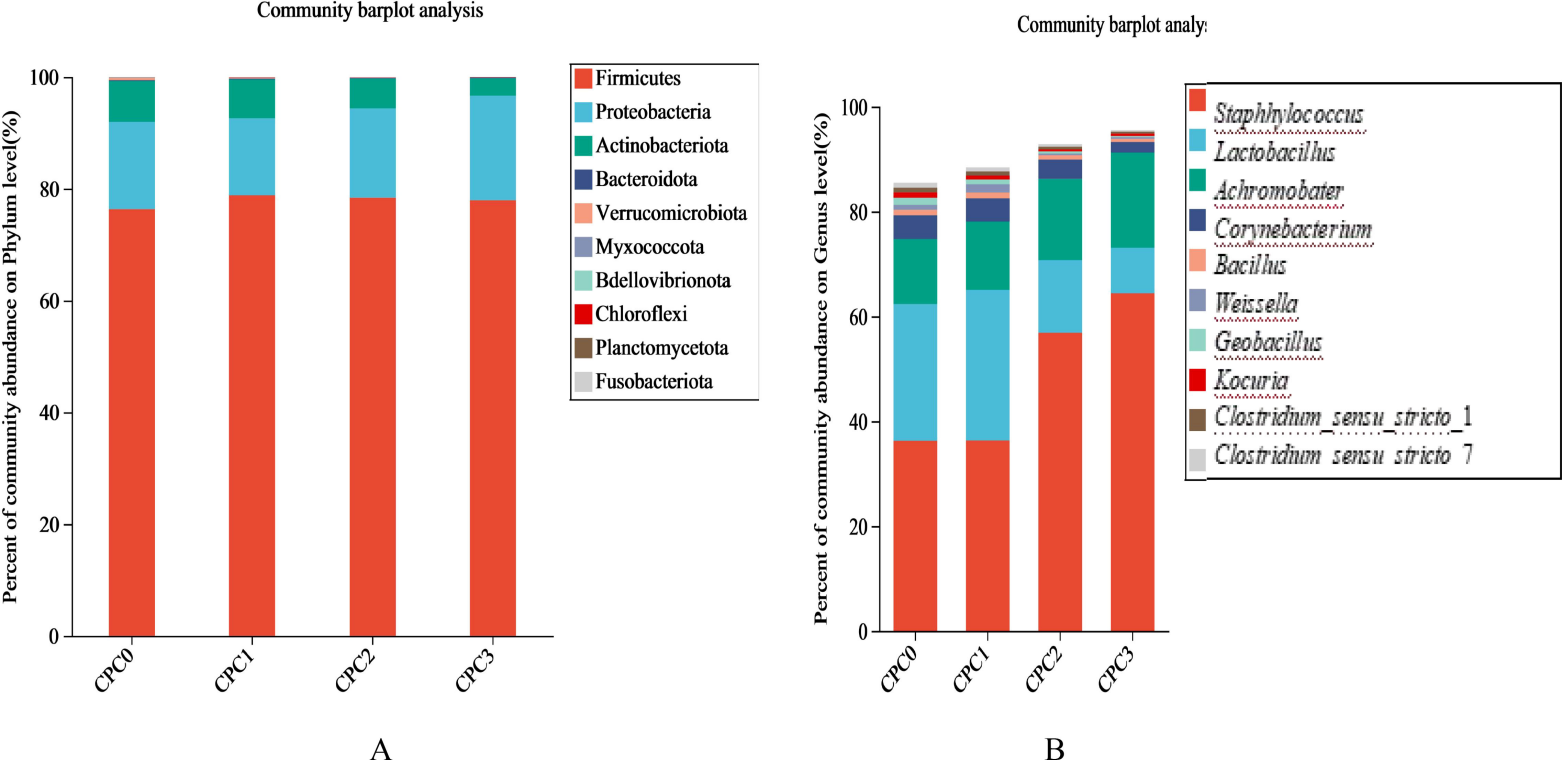
Community composition features of intestinal microflora in different dietary treatments. The abundance of the intestinal microbial composition at the phylum **(A)** and genus **(B)** levels in pikeperch (*Sander luciperca*) in the different groups after being fed for 8 weeks. Taxa with abundances <1% are included in “Others”.

At the phylum level, this study found that the top four phyla in the four groups were *Firmicutes*, *Proteobacteria*, *Actinobacteria*, and *Bacteroidetes* (**Figure 6A**; **Table 5**). Among the four phyla, the abundance of *Bacteroidetes* showed a downward trend, and the relative abundance of the CPC3 group was significantly lower than that of the other groups (*P* < 0.05). The F/B (*Firmicutes* to *Bacteroidetes* ratio) value first decreased and then increased with increasing dietary CPC levels, and the CPC3 group had the lowest F/B value (**Table 6**).

**Table 5:** Intestinal villi parameter of juvenile pikeperch fed different levels of cottonseed protein concentrate instead of fish meal.

Items	CPC0	CPC1	CPC2	CPC3
Villi height	320.47 ± 5.06^A^	272.84 ± 8.94^B^	352.71 ± 10.47^C^	383.21 ± 15.06^C^
Villi width	86.65 ± 5.65	90.08 ± 7.08	98.67 ± 6.83	107.05 ± 6.68
Layer thickness	58.95 ± 3.95^AB^	64.50 ± 5.01^AB^	70.00 ± 4.63^B^	51.41 ± 5.49^A^

**Table 6:** Relative abundance features of *Firmicutes*, *Proteobacteria*, *Actinobacteria*, and *Bacteroidetes* and the ratio of *Firmicutes*/*Bacteroidetes* in the intestine of pikeperch (*Sander luciperca*) under different treatments.

Item	CPC0	CPC1	CPC2	CPC3
*Firmicutes*/%	76.36 ± 3.32	78.83 ± 2.51	78.30 ± 2.87	77.84 ± 8.67
*Bacteroides*/%	0.14 ± 0.02	0.17 ± 0.05	0.13 ± 0.07	0.21 ± 0.08
F/B	592.98 ± 95.70	628.74 ± 149.89	771.96 ± 198.92	580.63 ± 148.03
*Proteobacteria*/%	15.62 ± 3.02	13.78 ± 2.27	15.98 ± 2.62	18.69 ± 8.00
*Actinobacteriota/*%	7.31 ± 0.66^b^	6.94 ± 0.24^b^	5.34 ± 1.00^b^	3.10 ± 0.70^a^

Among the values in each row, different capital letter suffixes represent the degree of data difference (*P* < 0.05), and n = 3.

At the genus level, the top four genera in the four groups were *Staphylococcus*, *Lactobacillus*, *Achromobacter*, and *Corynebaterium* (**Figure 6B**). As **Figure 6B** illustrates, the relative abundances of *Staphylococcus* and *Achromobacter* were statistically increased with increases in the dietary CPC substitution ratio, whereas the relative abundance of *Lactobacillu* and *Corynebaterium* were statistically reduced.

**Figure 7:**
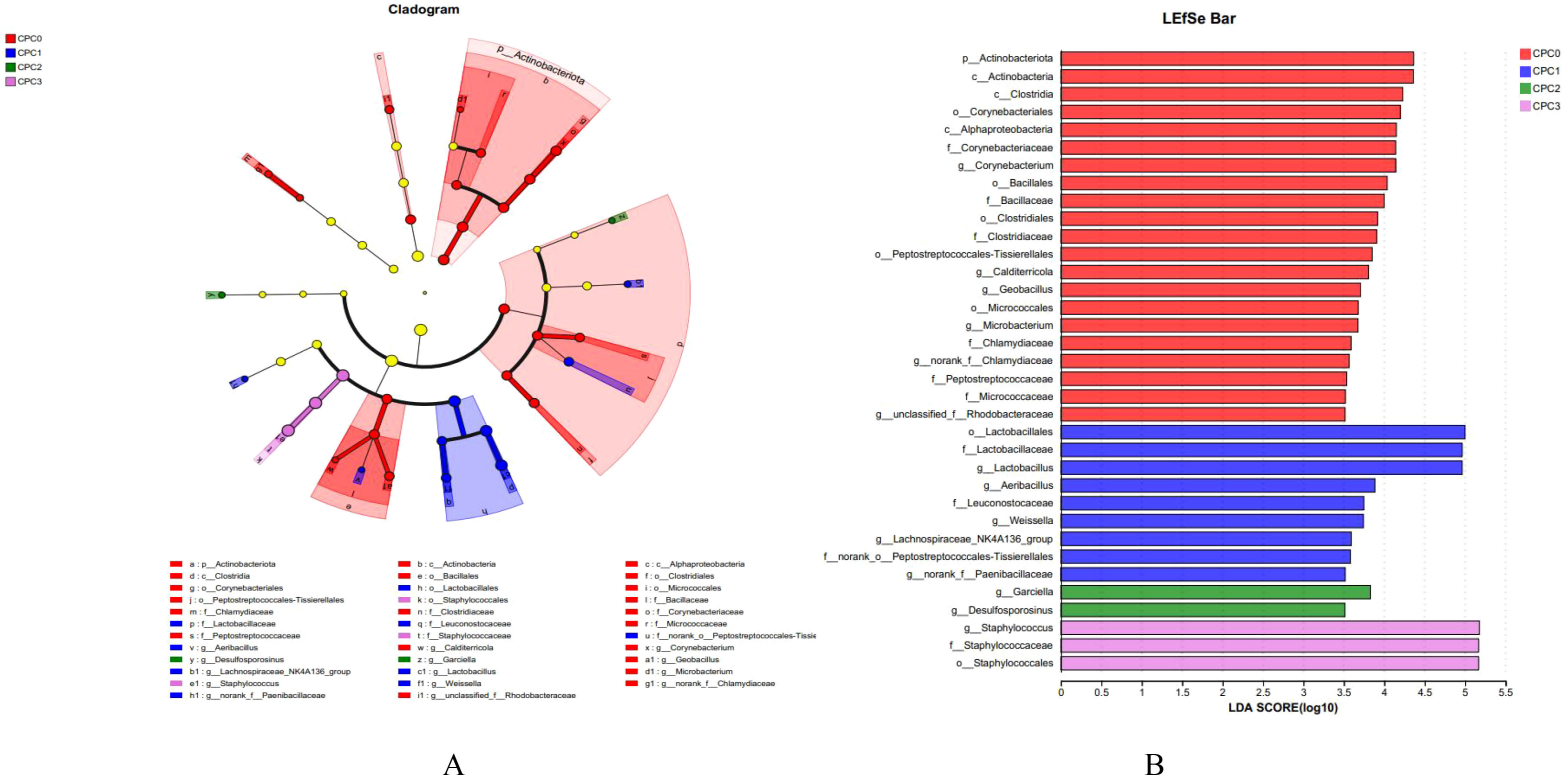
LEfSe analysis identified differential distributions of intestinal microbes among treatment groups. **(A)** Cladogram showing the phylogenetic distribution of intestinal microbes in the graded dietary protein level groups generated by the LEfSe analysis (layers of the cladogram represent different levels, phyla, classes, orders, families, and genera from the inside out). **(B)** LDA scores showed bacterial differential expression among the different treatments (LDA>3.0, *P* < 0.05).

LEfSe analysis indicated there were differences in the microflora taxa enriched in different dietary CPC substitution groups (**Figure 7**). CPC0 (red) was mainly enriched in *Actinbacteriota* and *Actinobacteria*. CPC1 (blue) was enriched in *Lactobacillates*, *Lactobacilacaae*, and *Lactobacillus*. The relative abundances of *Garciella* and *Desulfosporosinus* dominated in CPC2 (green). CPC3 (pink) was enriched in *Staphylococcus*, *Staphylococcaceae*, and *Staphylococcales*.

## 4 Discussion

### 4.1 Growth and feed utilization

Numerous research results have established an accepted fact that the substitution of fish meal with cottonseed protein concentrate will not adversely affect the growth and feed utilization of farmed carnivorous fish with a reasonable range of substitution (32, 33). In our study, there was no denying that replacing fish meal with CPC in pikeperch feed weakened the SR and growth indexes to some extent, but no significant differences were found between the CPC substitution groups and the control group. Unexpectedly, the 60% CPC substitution group obtained the most optimal MGR and SGR among the CPC substitution groups, indicating that the fish meal dosage, after CPC substitution, could be reduced to 22%. In a similar study on other farmed fish species in *Perciformes*, 50% CPC substitution reduced the fish meal level in sea perch feed (34) from 45% to 23% and in pearl gentian grouper (*Epinephelus fuscoguttatus*♀× *Epinephelus lanceolatus*♂) (35) feed from 50% to 25%. The results appear to suggest that CPC possesses the potential to substitute a relatively higher level of fish meal in the feed of farmed fish in *Perciformes*. The FER variation trend in our study also supports this conjecture. We found that the highest FER value was in the 60% CPC substitution group and it was significantly higher than that of the control group. Similar findings were published in a study of black sea bass (36), which reported that the lowest feed conversion rate (inverse of FER) occurred at the 80% CPC substitution level. Since the minimum dietary fish meal level designed in our experiment was 22%, it is not possible to determine whether cottonseed protein concentrate can be used as the main protein source to prepare fish meal-less or low-fish meal pikeperch feed. Further experiments are needed to confirm the potential of cottonseed protein concentrate as an alternative protein source for pikeperch.

### 4.2 Morphological index and composition of fish body

Studies have revealed that the application of CPC as a fish meal substitute did not significantly impact the morphology and composition of the bodies of black sea bass (36) and rainbow trout (37). However, other studies have demonstrated that replacing fish meal with CPC showed the opposite. Shen et al. (2019) reported that for *Trachinotus ovatus*, replacing up to 24% of the fish meal with CPC could significantly increase CF, and replacing up to 36% to 60% of fish meal with CPC could significantly increase the crude protein content. This change in morphology and composition of fish bodies also appears in our study. However, unlike previous studies that focused on significant changes in CF (38) and crude lipid content (19), our current study indicated that significantly lower HSI value and crude ash content and higher crude protein content were observed in the 60% CPC substitution group. A comparison of different studies may ascribe the differentiation to variations in cottonseed protein processing methods, fish species, and culture conditions.

### 4.3 Morphology and digestive enzyme activity of the intestine

The primary function of the intestine in fish is the digestion and absorption of foreign nutrients, which is directly embodied by morphology and digestive enzyme activity (39). Excellent morphological parameters including appropriate villus height, villus width, and muscle layer thickness can increase the digestive contact area and promote peristalsis (40). Fish digestive enzymes mainly include protease, lipase, and amylase. The activity of these enzymes reflects the digestive capacity of fish, affecting the absorption and utilization of protein, fat, and carbohydrate and the efficiency of energy acquisition (41). Studies on silver sillago (*Sillago sihama* Forsskál) (42) and large yellow croaker (20) have found that when CPC replaced a higher proportion of fish meal, the villus height, muscle thickness, and digestive enzyme activities tended to decrease. In our study, we also found that intestinal digestive enzymes including trypsin, lipase, and amylase presented a decreasing tendency, and, in particular, trypsin activity in the 60% substitution group was reduced to the lowest level compared to the control group and 20% substitution group. Previous research has indicated that replacing an overproportion of fish meal with plant protein sources will negatively affect the protease activity of the digestive organs of carnivorous fish (43, 44), This is probably due to a reduction in the bioactive substances with the increase in fish meal substitution which weakens the ability of the pancreas and intestinal glands to secrete digestive enzymes. Hence, decreased trypsin activity in the intestine might be an adaptation of juvenile pikeperch to concentrated cottonseed protein in the diet. However, unlike previous studies (20, 42), we found that the villus height in the intestine is significantly increased, suggesting that the intestine of pikeperch can adapt to an increase in CPC substitution by increasing peristaltic function.

### 4.4 Effect on antioxidative capacity and inflammation of the intestine

A higher proportion of plant protein can aggravate the oxidation stress of fish, thereby causing body damage and affecting the utilization efficiency of the plant protein (45). Concurrently, intestinal inflammation is triggered by the release of inflammatory factors due to oxidative stress (46). The change in antioxidant enzyme (SOD, CAT, etc.) activities and antioxidative substance content (GSH, etc.) could reflect the degree of oxidation to some extent (47). The intestinal immune system locally induces cells to produce pro-inflammatory factors such as interleukin-1 (*il-1*), *il-8*, and tumor necrosis factor-α (*tnfα*), and effector cells to produce anti-inflammatory factors such as transforming growth factor-β (tgf*-β*). Once the function and quantity of these two parts of cytokines are abnormal, intestinal homeostasis is broken, and the fish is extremely vulnerable to pathogen attack, which can induce a more intense inflammatory response in various tissues (48). Foodborne oxidative stress and inflammation caused by plant protein sources are generally attributed to their high content of anti-nutritional factors (49).

Evidentiary research has indicated that as anti-nutritional factors of cottonseed meal, excessive presence of gossypol in the aquatic feed could cause oxidation homeostasis in the liver or intestine and induce the secretion of inflammatory factors, thus resulting in intestinal inflammation (50–52). The origin of cottonseed protein concentrate is precisely derived from the removal of anti-nutritive gossypol from cottonseed meal (16). Research on largemouth bass found that when the gossypol content of CPC was optimized to 169.36 mg/kg, a 30% substitution of fish meal with gossypol-reduced CPC could significantly increase liver antioxidant activity, upregulate the mRNA expression of liver anti-inflammatory factors, and downregulate the mRNA expression of pro-inflammatory factors compared to the no-gossypol control group (53). Nonetheless, excess CPC in the feed caused the fish intestine to exhibit an inflammatory response (26, 54). Our current results showed that when the gossypol content of the CPC was reduced to 230 mg/kg, the 20% to 60% substitution of fish meal with gossypol-reduced CPC still significantly decreased the intestinal activities of CAT and SOD and contents of GSH and T-AOC, downregulated the mRNA expression of *tgf-β*, and upregulated the mRNA expression of pro-inflammatory factors (*il-1β*, *il-8*, and *tnf-α*) compared to the control group. This finding may imply that the tolerance of pikeperch to gossypol is relatively weaker than that of largemouth bass to a certain extent. It is noteworthy that among the three CPC substitution groups, increases in the intestinal antioxidant enzyme activities, GSH content, and anti-inflammatory factor expression and downregulation in pro-inflammatory factors were observed with increasing CPC substitution ratios. Combined with existing studies, we speculate that increased contents of prebiotics (such as raffinose and stachyose) with the addition of increasing CPC could partially counteract the negative effects of gossypol (18, 55). In any case, the specific mechanism needs to be further explored.

### 4.5 Effect on the mechanical barrier of the intestine

The intestinal mechanical barrier is a vital defense line against the invasion of pathogens and is mainly composed of epithelial cells and intercellular connections (56). Mechanical damage of intestinal mucosa caused by plant protein sources has been demonstrated in many fishes (31, 57). The intestinal villus height of *Cyclopterus lumpus* was shortened and the lamina propria width was increased when a mixture of soybean protein concentrate and pea protein concentrate replaced more than 25% of fish meal (58). In the current study, when 20% to 60% fish meal was replaced by CPC, the intestinal mucosa of pikeperch presented with mechanical damage in comparison with the control group, which was characterized by uneven intestinal wall thickness and short and sparse intestinal villi. Similar findings were found in a study on largemouth bass (15), which reported that excessive replacement of fish meal with CPC impaired intestinal structure. The permeability of intestinal tight junctions determines the barrier function of the entire intestinal epithelial cells. Transmembrane proteins, such as occludin and claudins, and peripheral cytoplasmal proteins, such as Zos, form a narrow band structure from the outside to the inside, sealing the cell space and preventing intestinal toxic substances from entering the surrounding tissues (59). Merrifield et al. (60) found that the exposure of intestinal cells to tight junctions was correlated with the height and density of intestinal villi. Analogously, CPC substitution levels of 20% to 60% significantly downregulated the gene expression of *occludin-a*, *zo-2*, and *claudin-15a* compared to the control group, which was consistent with changes in intestinal morphological damage. In accordance with the study in our previous study, the morphology and barrier function of common carp (*Cyprinus carpio*) intestines were damaged when the amount of CPC replacing fish meal exceeded 50% (61). Notably, the injury degree of the intestinal mechanical barrier was mitigated along with relief from oxidative damage and inflammation with increasing CPC substitution ratios. The adaption mechanism needs to be further explored in the future.

### 4.6 Effect on the biological barrier of the intestine

The intestinal biological barrier is mainly composed of normal intestinal flora, which can promote the proliferation of intestinal epithelial cells and strengthen the immune function of fish (9, 62–66). Diverse research studies have pointed out that CPC application in feed directly differentiated intestinal microflora structure in aquatic animals (38, 61, 67). Our study on pikeperch indicated that CPC substitution levels of 20% to 60% distinctly changed the intestinal microflora composition and lessened the bacterial diversity and richness, as shown by the Shannon, ACE, chao, and Simpson indices. The NMDS analysis further revealed that the degree of separation of microflora composition tended to be more obvious with increasing dietary CPC substitution ratios. These results suggest that the dietary CPC substitution ratio could vary the intestinal microflora structure of pikeperch.

Our results revealed that the abundances of the phyla *Firmicutes*, *Proteobacteria*, and *Actinobacteriota* occupied the first three positions in the microflora community of pikeperch following *Bacteroidota*, which was in line with our previous study on the intestinal microflora of pikeperch (9). In this study, increasing the dietary CPC substitution ratio slightly increased *Firmicutes* abundance and significantly decreased *Actinobacteriota* abundance, but the abundance of *Proteobacteria* in the 60% substitution group was appreciably increased. Similar results were also observed in the study on golden pompano (18) and grass carp (*Ctenopharyngodon idellu*s) (38). Because an excess of *Proteobacteria* in the intestine could lead to metabolic disturbances or inflammation (68), an abnormal increase in *Proteobacteria* abundance may have been a potential risk of CPC being used to replace fish meal in pikeperch feed. Apart from this, our study examined the abundance ratio (F/B ratio) of *Firmicutes* and *Bacteroides*. Variation of the F/B ratio can indirectly reflect the metabolic homeostasis and growth state (68). In this study, the F/B value of the 60% CPC substitution group appreciably decreased and was positively correlated with a reduction in final mass and MGR. This suggests that CPC has the potential to promote growth by improving the intestine microflora structure.

At the genus level, *Staphylococcus* and *Lactobacillus*, belonging to *Firmicutes*, and *Achromobacter*, belonging to *Proteobacteria*, were the most dominant bacteria genera in our study. Among these, *Staphylococcus* and *Achromobacter* were the main bacteria genera in the 60% substitution group, and the abundance of the two genera increased gradually with the increase in CPC substitution. *Staphylococcus*, among gram-positive bacteria, is the most important infection pathogen (69). Similarly, an increased abundance of *Staphylococcus* was associated with intestinal disorder in common carp (70). In addition, a considerable decrease in *Lactobacillus* abundance was detected in the 40% and 60% substitution groups compared to the control group and 20% substitution group. It has been reported that *Lactobacillus* can regulate the homeostasis of the intestinal microecological environment and change the structure of the host’s intestinal microflora, thus improving the intestinal barrier defense against bacterial diseases (71). Together, the increase in *Staphylococcus* and the decrease in *Lactobacillus* caused by exorbitant CPC substitution might be deemed an ignition point for damage to intestinal microflora homeostasis and immune ability in this study.

### 4.7 Genes associated with the GH/IGF axis and TOR signaling pathway in the intestine

The GH/IGF-1 axis has been regarded as an effective tool to regulate the growth of fish (72). Hence, variation genes in the GH/IGF-1 axis were mapped with changes in growth performance. As reported in a study on Nile tilapia (*Oreochromis niloticus*), 28 days of starvation suppressed growth and CF, with a concomitant decline in the expression level of IGF-1 (73). The nutrient composition of feed can also regulate the GH/IGF axis, thereby affecting the growth of fish (74). Evidential research has revealed that the level and source of dietary protein or lipids are tightly linked to GH and IGF-I or their mRNAs in gilthead sea bream (*Sparus aurata* L.) (75), Nile tilapia (76), and common carp (77). It was further shown that the utilization of alternative plant proteins in fish feed may result in liver GH desensitization and inhibit GHR and liver IGF-I gene expression. For instance, the expression of *ghr* and *igf1* genes in the livers of common carp bream was significantly decreased after partial or total replacement of fish meal by CPC in common carp feed (61). The findings of the current study indicated that the expressions of *ghr* and *igf2* first decreased and then rose with increasing CPC replacement levels in diets, which was basically consistent with the changing trend of the growth indexes. However, *igf1* expression declined gradually with increasing CPC replacement levels in the diets. Furthermore, the GH/IGF axis also interferes with the nutritional status of tissues by regulating downstream pathways. Therein, activation of the IGF-phosphoinositol triphosphate (PI3K)-Akt-rapamycin target protein (mTOR) metabolic pathway during growth can effectively enhance protein synthesis in cells (78). To regulate protein metabolism in response to various nutrient quality and environmental conditions, activated TOR can regulate two parallel signaling pathways (4E-BP and S6K) to coordinate the balance between protein synthesis and protein degradation (79). Previous studies in the literature on grass carp (38) and common carp (61) reported that high levels of dietary CPC replacing fish meal depressed protein synthesis by inhibiting the activation of the TOR signaling pathway. However, this study found that the expression of *mtor*, *akt1*, *rps6k1*, and *eif4ebp* first decreased and then rose as the dietary CPC supplement level increased. Notably, the expressions of the four genes in the 60% substitution group were markedly enhanced compared to those in the 20% and 40% substitution groups. This change was very similar to the trend of crude protein content in the whole fish, which may be due to the fact that activation or shutdown of the TOR signaling pathway positively feeds back into protein synthesis. Meanwhile, this study also established that the impaired protein synthesis caused by the weakening of the TOR pathway is triggered by the decreased expression of *igf1*. Overall, these findings seem to imply that IGF1 in the GH/IGF axis and the IGF-PI3K-Akt-mTOR pathway is a key breakthrough in improving the growth-promoting effectiveness of CPC when replacing fish meal for pikeperch.

## 5 Conclusions and future perspectives

In summary, this study systematically investigated the intestinal response characteristics of immune function and microflora to CPC as a fish meal alternative in pikeperch. The present study on pikeperch showed that replacing 60% of dietary fish meal with CPC showed a higher application potential in comparison with 20% and 40% as evidenced by the optimal growth performance, stronger intestinal antioxidant capacity, healthier intestinal morphology, and more solid intestinal mechanical barrier, although the above indices in the 60% substitution group were still weaker than the control group. An in-depth analysis revealed that there were key factors leading to the poor use of CPC. First, the elevation of *Staphylococcus* and the reduction of *Lactobacillus* caused by exorbitant CPC substitution disturbed the homeostasis of the intestinal microecological, thereby damaging the intestinal microflora homeostasis and immune ability. Second, decreased expression of *igf1* with increasing CPC substitution level impaired protein synthesis and thus caused a decrease in protein deposition. The current findings provide a signficant reference and breakthrough in improving the effectiveness of replacing fish meal with CPC in pikeperch diets.

## Data Availability

The original contributions presented in the study are publicly available. This data can be found here: National Center for Biotechnology Information (NCBI) BioProject database under accession number PRJNA1227703, BioSample accessions SAMN46990986-SAMN46990997 and This data can be found here: https://www.ncbi.nlm.nih.gov/bioproject/PRJNA1227703.
