# Supplementary material for: Cottonseed protein concentrate as an effective substitute to fish meal in pike perch (Sander luciperca) feed: evidence from growth performance and intestinal responses of immune function and microflora
Source: Front Immunol. 2025 Mar 4;16:1522005. doi: 10.3389/fimmu.2025.1522005 (PMC11920712; doi:10.3389/fimmu.2025.1522005)
Supplement: Supplementary file 1 [file DataSheet1.docx]

| 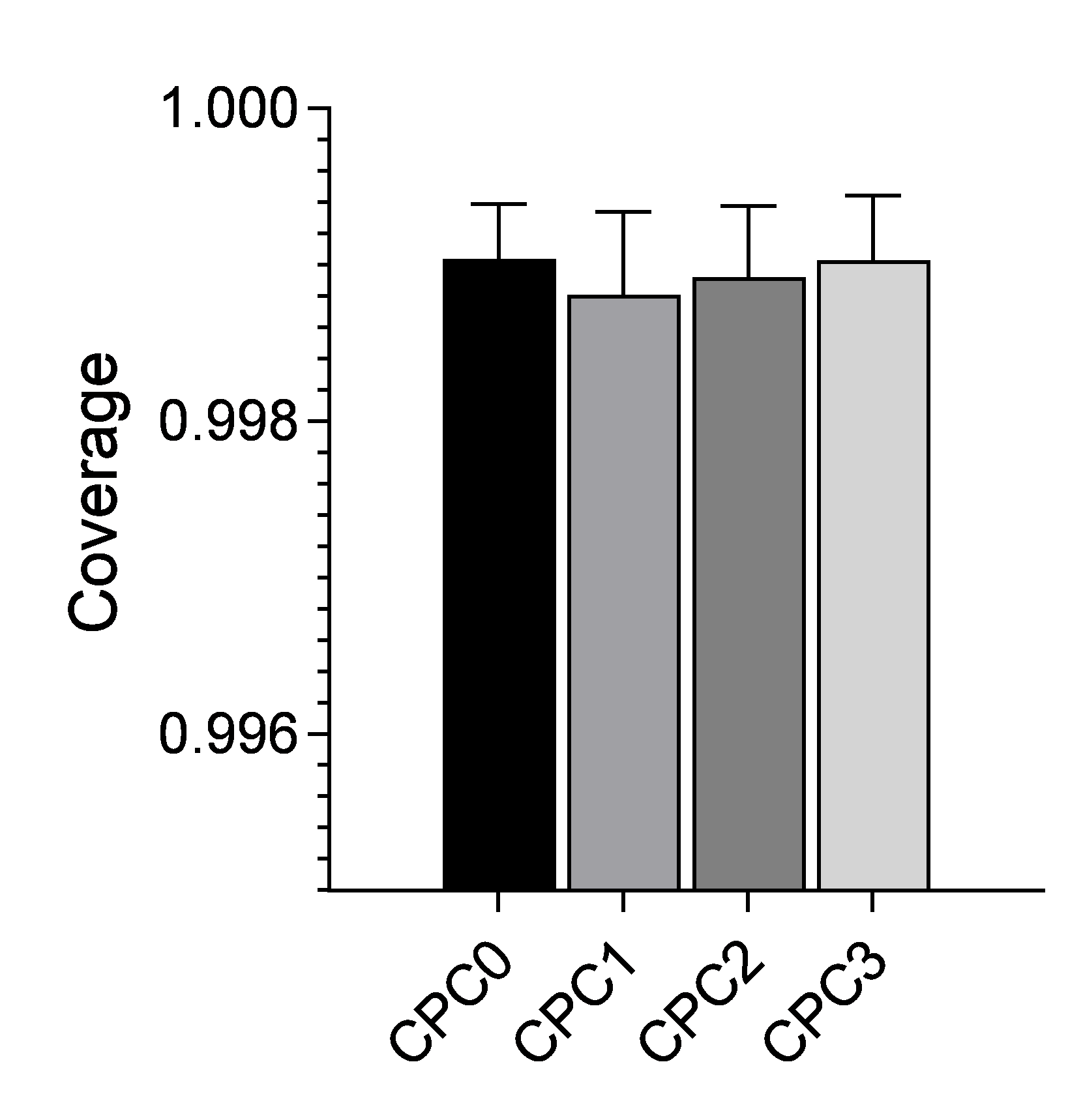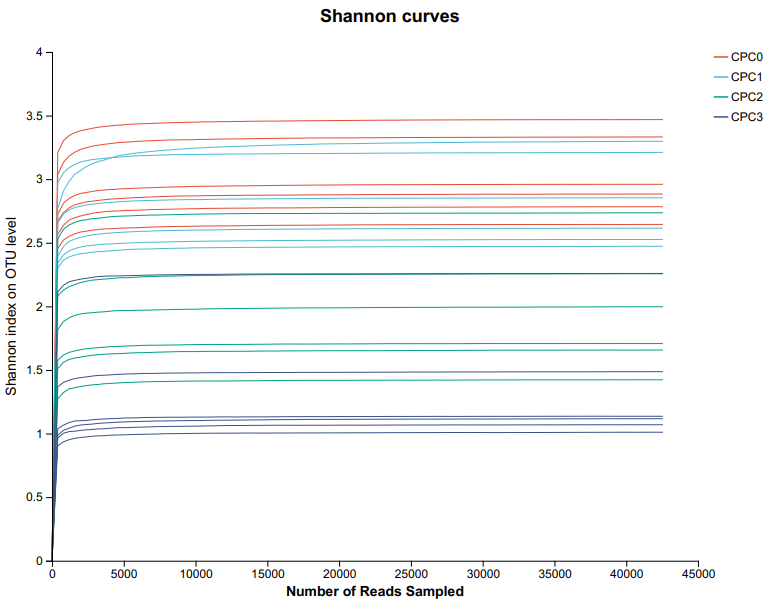A. Shannon curves B. Coverage index |
| --- |
| Fig. S1 Shannon curves and coverage index in Alpha diversity analysis of intestinal microflora in pikeperch (*Sander luciperca*) fed with increasing substitution ratio of cottonseed protein concentrate (CPC). |
